# Blood pressure and kidney function in neonates and young infants with intrauterine growth restriction

**DOI:** 10.1007/s00467-022-05713-z

**Published:** 2022-09-02

**Authors:** Katharina Monika Heuchel, Fabian Ebach, Ebru Aileen Alsat, Heiko Reutter, Andreas Mueller, Alina Christine Hilger

**Affiliations:** 1 Faculty of Medicine, University Bonn, Bonn, Germany; 2 Department of Neonatology and Pediatric Intensive Care, University Hospital Bonn, Bonn, Germany; 3 Division of Neonatology and Pediatric Intensive Care, Department of Pediatrics and Adolescent Medicine, Friedrich-Alexander University Nürnberg-Erlangen, Erlangen, Germany; 4 Department of Pediatrics and Adolescent Medicine, Friedrich-Alexander University Nürnberg-Erlangen, Erlangen, Germany; 5 Research Center On Rare Kidney Diseases (RECORD), University Hospital Erlangen, Erlangen, Germany

**Keywords:** Intrauterine growth restriction, Proteinuria, Kidney function, Blood pressure, Neonates

## Abstract

**Background:**

Intrauterine growth restriction (IUGR) has been associated with changes in kidney anatomy, nephrogenesis and the vascular system, resulting in secondary arterial hypertension and kidney damage in adulthood. Here, we compare routine clinical and metabolic parameters between IUGR and non-IUGR study participants in the neonatal and early infant period.

**Methods:**

A total of 39 IUGR and 60 non-IUGR neonates were included during an 18-month study period. We compared blood pressure, serum creatinine (SCr), urea nitrogen (BUN), urinary albumin, α-1-microglobulin, transferrin, immunoglobulin G and total protein excretion in spontaneous urine normalized by urine creatinine level during the hospital stay.

**Results:**

There were no significant differences in mean values of blood pressure and urinary protein excretion between cases and controls. SCr and BUN levels were lower in the IUGR group compared to the non-IUGR group.

**Conclusions:**

The lower levels of SCr and BUN may be attributed to lower liver and muscle mass in IUGR neonates and young infants. Biomarkers currently used in routine clinical care do not allow early postnatal prediction of higher blood pressure or worse kidney function due to IUGR, so further studies are needed.

**Graphical abstract:**

A higher resolution version of the Graphical abstract is available as Supplementary information.

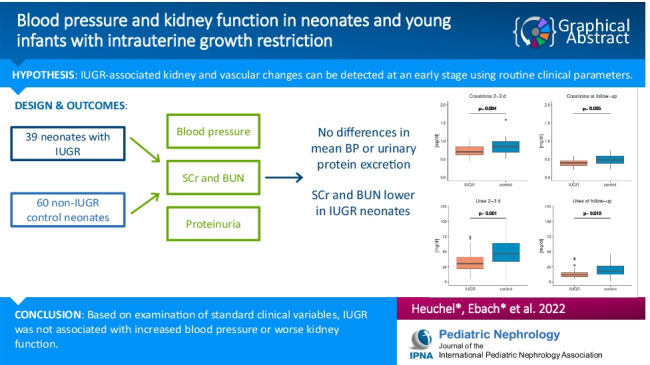

**Supplementary Information:**

The online version contains supplementary material available at 10.1007/s00467-022-05713-z.

## Introduction

Intrauterine or fetal growth restriction (IUGR/FGR) is defined by the inability of a fetus to reach its genetically predetermined growth potential due to an underlying pathology and occurs in approximately 5–10% of all pregnancies [1, 2]. Causes are heterogeneous including maternal, placental and/or fetal factors [3, 4]. IUGR definition may vary between countries or medical organisations. Most guidelines define IUGR as an estimated fetal weight below the 10th percentile and/or a decrease in growth velocity if there is also a pathological finding in Doppler sonography of the umbilical or uterine artery [4–7]. In other definitions, an estimated fetal weight below the third percentile is often considered to be sufficient for the diagnosis of IUGR [5–7]. IUGR has been associated with higher mortality, higher morbidity including, for example, nutritional disorders, septitides and respiratory distress syndrome, as well as with adult-onset diseases such as obesity, diabetes mellitus or cardiovascular disease [8–13]. Moreover, in human and animal studies, IUGR has been associated with changes in kidney anatomy, nephrogenesis and the vascular system [14–16]. In particular, IUGR may be related to an increased frequency of arterial hypertension and kidney damage in adulthood [14, 17–19]. Human post-mortem as well as murine studies have shown that IUGR is associated with a reduced number of glomeruli compared to non-IUGR controls [16, 18, 20]. Nevertheless, these observations are still subjects of ongoing research. The aim of our prospective cohort study was to identify possible IUGR-related abnormalities in routine clinical parameters such as blood pressure, proteinuria and kidney function in the early postnatal period.

## Methods

### Study design and patients

IUGR neonates admitted to our neonatal intensive care unit (NICU) or to our newborn nursery at the University Hospital Bonn between April 2019 and September 2020 were eligible for study participation if they remained hospitalised in our NICU and/or newborn nursery for at least 72 h after birth and prenatal findings met the German, Austrian and Swiss 2017 consensus guideline for IUGR (“fetal estimated weight < 10th percentile and/or non-percentile growth during the progress and pathological Doppler sonography of the umbilical or uterine artery or oligohydramnion”) [4]. The study period ended with discharge from the hospital. All neonates admitted to our NICU and newborn nursery during the same period, who did not meet the criteria for IUGR with a birth weight between the 10th and the 90th percentile and who remained hospitalised in our NICU and/or newborn nursery for at least 72 h after birth, were eligible to be enrolled as controls. Exclusion criteria for cases and controls were chromosomal anomalies, congenital malformations of the kidneys or urinary tract, haemodynamically relevant congenital heart defects, congenital diaphragmatic hernia (CDH), the need for extracorporeal membrane oxygenation (ECMO) therapy, unstable condition according to the assessment of the treating physicians, perinatal asphyxia or death during hospitalisation. Informed written consent was obtained from parents or legal representatives before enrolment in the study. This study was approved by the local Ethics Committee of the University of Bonn Medical School (22.05.2020/No. 079/19).

### Clinical evaluation

We retrospectively extracted the following information from the mothers’ and children’s patient records: sex, birth weight, gestational age, length at birth, APGAR scores, pH from the umbilical artery, occurrence of birth complications, birth mode, maternal age, gravida, body mass index, maternal morbidity, medication during pregnancy, drug abuse in pregnancy, single or multiple pregnancy, pregnancy complications, blood pressure levels, protein intake, medication, drug levels in serum when taking nephrotoxic drugs (vancomycin, tobramycin), body weight and length on discharge and duration of hospitalisation.

### Blood pressure measurements

Both invasive and non-invasive blood pressure measurements were considered. Systolic, diastolic and mean arterial pressures were calculated as means of individual measurements during a predefined period of time (BP1: between 36th and 72nd hour of life, BP2: 8th day of life, BP3: last five measurements before discharge). Since the time of discharge significantly differed between cases, there is a large variability in timing of BP3 (between 3rd and 161st day of life). In cases where children were discharged before the 8th day of life (10/99), no BP2 could be registered and the blood pressure before discharge was registered (BP3). In order to control for patient’s age and gestational age, we compared the blood pressure measurements to the patients’ individual reference values obtained from Pejovic et al. [21].

### Laboratory testing

**Fig. 1:**
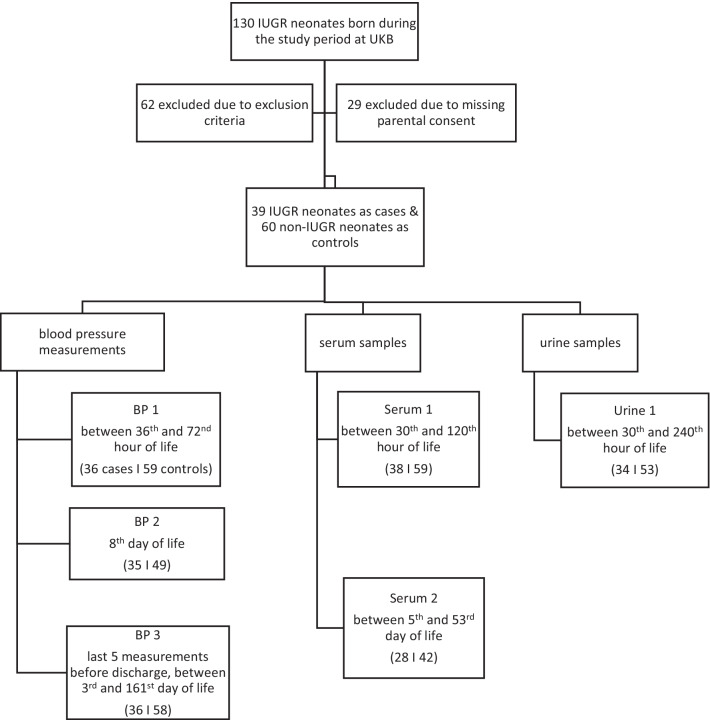
Flow chart of the included study participants, recorded laboratory values and blood pressure measurements, as well as the defined time intervals. IUGR, intrauterine growth restriction; BP, blood pressure

Serum creatinine (SCr) and blood urea (BUN) levels were measured only in the context of medically necessary blood sampling. The first measurement was between the 30th and 120th hour of life (serum 1), mostly at the time of the newborn screening (between 36th and 72nd hour of life). Also, a second blood sample was obtained only when a blood draw was medically indicated (e.g. repeat newborn screening in preterm neonates or infants) and therefore shows a high variability with regard to timing (between 5th and 53rd day of life) (serum 2). We compared the measured SCr and BUN values with corresponding reference values [22, 23] to investigate whether one group (IUGR or controls) exceeded the limits more frequently than the other. Urine was collected regardless of clinical indication with consent of the legal guardians between the 30th and 240th hour of life. Non-invasive measures such as urine bags or insertion of compresses into the diaper were used to obtain spontaneous urine unless a urinary catheter was medically indicated. To detect proteinuria, we measured albumin, α-1-microglobulin, transferrin, immunoglobulin G and total protein in spontaneous urine and normalized the measured values by urine creatinine level. We compared the measured urine excretion of the listed parameters with respective specific limits [24–26], except for transferrin, for which no neonatal reference values are available, to again investigate whether one group (IUGR or controls) exceeded the limits more frequently than the other. All collected blood and urine samples were processed immediately at the central laboratory facility of the University Hospital Bonn. SCr, urine creatinine and BUN levels were measured using VIS photometry on a cobas c702 analyser with the reagent CREJ2 for creatinine and UREAL for urea (Roche Diagnostics). Albumin, immunoglobulin G and total protein were measured using turbidimetry with the specific reagent on a cobas c702. Turbidimetry was also used to measure α-1-microglobulin levels but on a cobas c502. To determine the transferrin content, immunnephelometry was used on a BN ProSpec. If a value was below the respective detection limit, calculations were performed using a constant value below that of the lower limit. The respective limits of detection (LODs) in the urine and values used for further calculation are listed here: creatinine: LOD 4.2 mg/dl, used constant value 4.1 mg/dl; albumin: LOD 3 mg/l, used constant value 2.9 mg/l; α-1-microglobulin: LOD 5 mg/l, used constant value 4.9 mg/l; transferrin: LOD 2.3 mg/l, used constant value 2.2 mg/l; and total protein: LOD 40 mg/l, used constant value 39.9 mg/l. In one case, the upper detection limit of α-1-microglobulin (LOD 400 mg/l) was exceeded; here, we calculated with a constant value (401 mg/l) above the detectable range. An overview of the recorded laboratory values is shown in Fig. 1.

### Protein intake

During inpatient treatment, the protein intake of all patients in our department is automatically calculated by our medical documentation system. To exclude protein intake as a major confounder influence of BUN concentration, we extracted the respective intakes at both times of serum sampling from the individual patient’s medical record and compared it between cases and controls.

### Statistical analysis

Statistical analyses were performed using R version 3.6.2 [27]. Comparisons of the discrete data of both groups, IUGR and non-IUGR neonates, were conducted using Pearson’s chi-squared test or Fisher’s exact test in case of expected cell counts below 5 in the respective contingency table. Student’s *t* test and Mann–Whitney *U* test for normally distributed and non-normally distributed data, respectively, were applied to the collected metric data. The Shapiro–Wilk test with an error level of *p* = 0.01 was used to check whether there was a normal distribution for the collected data. Starting from an alpha error of 5% for a two-sided test, Bonferroni correction was applied to neutralise the accumulation of alpha errors in multiple comparisons. Due to the variable timing of the second serum analysis (together with a medically indicated blood sampling) and BP3 (prior to discharge), we performed an analysis of covariance (ANCOVA) to control for timing as a confounder. Percentile and *z* scores were adapted from Voigt et al. [28].

## Results

### Study population

During the period of our study, 130 neonates were born with IUGR in the University Hospital Bonn. Of these 130 neonates, 62 were not eligible for inclusion because they met at least one of the exclusion criteria. An additional 29 IUGR neonates could not be included as there was no parental consent for participation in the study (Fig. 1). These neonates did not differ significantly from participants in gestational age, birth weight or sex.

Of the neonates participating in the study, 39 were enrolled in the IUGR case group with an average gestational age of 31.90 weeks (standard deviation (SD) 3.46 weeks). Sixty neonates were enrolled in the control group, with an average gestational age of 32.45 weeks (SD 2.91 weeks). As entailed by the definition of IUGR, birth weight was significantly lower in the IUGR group 1.38 kg (SD 0.52 kg) than in the control group 1.96 kg (SD 0.58 kg) (mean *z* scores: IUGR group − 1.43, SD 0.58; control group − 0.08, SD 0.58). Consecutively, birth length (39.23 cm, SD 5.12 cm; mean *z* score − 1.33, SD 0.74) in the IUGR group was significantly lower than in the control group (43.83 cm, SD 4.48 cm; mean *z* score − 0.02, SD 0.88). The mean weight-for-length *z* scores were − 3.01 (SD 1.92) in the IUGR group and 0.23 (SD 2.36) in the control group. Expectedly, body weight at discharge was still lower in IUGR patients (2.08 kg, SD 0.28 kg; mean *z* score − 2.53, SD 0.66) than in control patients (2.39 kg, SD 0.32 kg; mean *z* score − 1.40, SD 0.60, *p* < 0.001). The same was true for body length at discharge (IUGR 43.64 cm, SD 1.97 cm; mean *z* score − 2.96, SD 0.93; controls 46.74 cm, SD 2.85 cm; mean *z* score − 1.22, SD 1.30; corrected *p* < 0.001). *Z* scores at discharge are based on the corrected gestational age. Other demographic data did not differ (see Table 1). Neonates in the IUGR group were more likely to be treated with blood pressure-increasing drugs like catecholamines and glucocorticoids at the time of the first measurement (BP1) than neonates in the control group. This difference was not significant after Bonferroni correction for multiple comparisons (*p* = 0.039). The application of the first line antibiotic tobramycin was comparable in both groups, whereas the IUGR cases received second-line antibiotic treatment with vancomycin non-significantly (*p* = 0.039) more frequently during hospitalisation. In every patient, vancomycin and tobramycin were monitored by serum-level controls, and none of the levels measured reached the nephrotoxic range (tobramycin > 12 µg/ml, vancomycin > 30 µg/ml). There was no acute kidney injury (AKI, based on neonatal AKI KDIGO classification [29]) observed in the participants of the IUGR or the control group. The length of hospitalisation and hence the study period differed between cases and controls, with a median hospital stay of 31 days for IUGR cases compared to 19 days for controls.

**Table 1:** Clinical characteristics of cases and controls. If data were normally distributed, standard deviation is given and *p* refers to a *t* test comparison of means. If data was non-normally distributed, interquartile ranges are provided and *p* refers to a Mann–Whitney *U* test. *Z* scores at discharge refer to the corrected gestational age

	IUGR (*n* = 39)	Control (*n* = 60)	*P* overall
Male	17 (44%)	28 (47%)	0.925
Birth weight (kg)	1.38 ± 0.52*	1.96 ± 0.58*	< 0.001
Mean *z* score	− 1.43 ± 0.58*	− 0.08 ± 0.58*	
Birth length (cm)	39.23 ± 5.12*	43.83 ± 4.48*	< 0.001
Mean *z* score	− 1.33 ± 0.74*	− 0.02 ± 0.88*	
Weight-for-length (g/cm)	34.23 ± 9.66*	44.01 ± 9.95*	
Mean *z* score	− 3.01 ± 1.92*	0.23 ± 2.36*	
Gestational age (weeks)	31.90 ± 3.46*	32.45 ± 2.91*	0.416
Umbilical artery pH	7.34 [7.27; 7.38]**	7.36 [7.28; 7.39]**	0.376
Treatment with nephrotoxic drugs			
Vancomycin	10 (26%)	5 (8%)	0.039
Tobramycin	16 (41%)	21 (35%)	0.694
Treatment with BP-modifying drugs			
BP-increasing 36th–72nd hour of life	10 (26%)	5 (8%)	0.039
BP-decreasing 36th–72nd hour of life	14 (36%)	16 (27%)	0.452
BP-increasing 8th day of life	3 (8%)	2 (3%)	0.380***
BP-decreasing 8th day of life	3 (8%)	7 (12%)	0.736***
BP-decreasing before discharge	3 (8%)	2 (3%)	0.380***
Body weight at discharge (kg)	2.08 ± 0.28*	2.39 ± 0.32*	< 0.001
Mean *z* score	− 2.53 ± 0.66*	− 1.40 ± 0.60*	
Body length at discharge (cm)	43.64 ± 1.97*	46.74 ± 2.85*	< 0.001
Mean *z* score	− 2.96 ± 0.93*	− 1.22 ± 1.30*	
Length of hospitalisation (days)	31 [19.5; 74]**	19 [12.75; 37.25]**	0.0486
Maternal age (years)	30.9 ± 5.5*	32.9 ± 4.6*	0.069
Maternal BMI (kg/m^2^)	24.4 [20.9; 27.9]**	23.9 [21.5; 27.3]**	0.969
Gravida			0.810
1	17 (44%)	26 (43%)	
2	11 (28%)	14 (23%)	
≥ 3	11 (28%)	20 (33%)	
Multiple pregnancy	15 (38%)	31 (52%)	0.280
Vaginal delivery	5 (13%)	14 (23%)	0.300
Drug abuse in pregnancy	8 (21%)	4 (7%)	0.081***
Induced lung maturation	23 (59%)	36 (60.0%)	1.000

BP-increasing drugs: glucocorticoids and catecholamines. BP-decreasing drugs: diuretics, opioids, β-blocker, glycerol trinitrate, milrinone, urapidil, levosimendan, clonidine, amlodipine, sildenafil, iloprost and chloral hydrate

*IUGR* intrauterine growth restriction, *BP* blood pressure, *BMI* body mass index

^*^Standard deviation

^**^Interquartile range

^***^In case of expected frequencies, < 5 Fisher’s exact test was applied

### Blood pressure measurements

**Fig. 2:**
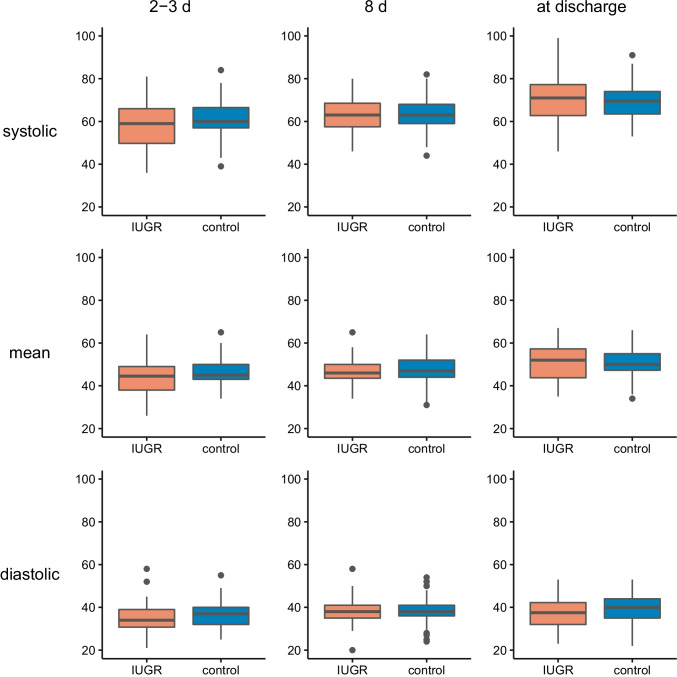
Arterial blood pressures at different time points. Box plots indicate median (black bar), upper and lower IQR (hinges) and the upper and lowermost point not exceeding 1.5 × IQR (whiskers). Outliers are marked as black points. IUGR, intrauterine growth restriction

Figure 1 displays the data available for each time point of blood pressure measurement. There was no significant difference in blood pressure between cases and controls at any time (Online Resource 1). Compared with their respective normal values, IUGR patients did not exceed *(χ*^2^ = 0.19, *p* = 0.66) or fall below *(χ*^2^ = 0, *p* = 1) their normal range obtained from Pejovic et al. [21] more often than control patients did. To account for the large variability in the timing of BP3 (prior to discharge), we performed an analysis of covariance controlling for measurement timing as a confounder. Again, we found no significant effect of IUGR on systolic (*p* = 0.82), mean (*p* = 0.24) or diastolic (*p* = 0.16) blood pressure values in BP3. Blood pressure measurements for both groups are comparatively visualized in Fig. 2.

### Kidney function

**Fig. 3:**
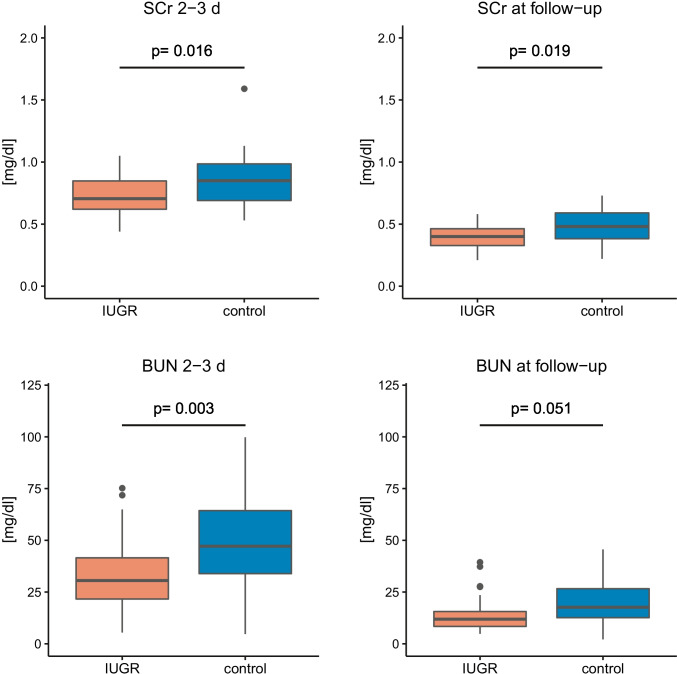
SCr and BUN levels. First measurement (serum 1) at blood collection between 30th and 120th hour of life. Follow-up measurement (serum 2) with routine blood sampling between 5th and 53rd day of life. The *p* values displayed are Bonferroni-corrected and refer to a Mann–Whitney *U* test comparing IUGR and control patients. Box plots indicate median (black bar), upper and lower IQR (hinges) and the upper and lowermost point not exceeding 1.5 × IQR (whiskers). Outliers are marked as black points. IUGR, intrauterine growth restriction; SCr, serum creatinine; BUN, blood urea nitrogen

In total, 97/99 (38/39 IUGR, 59/60 control) neonates had measurement data available at the first measuring time (serum 1). In 81 of these 97 neonates (29 IUGR, 52 control), a serum sample could be obtained between the 36th and 72nd hour of life. The second blood sampling (serum 2) could be obtained at 16.5 days of life (median) (interquartile range (IQR) 11.00 − 25.75 days, min 5 days, max 53 days) in 28/39 IUGR cases and 42/60 controls. In 34 study participants (14/39 IUGR, 20/60 control), the second measurement (serum 2) was taken as part of the repeat newborn screening with a corrected gestational age of 32.0 weeks. At both times of blood sampling, SCr levels were lower in the IUGR group (serum 1 0.71 [IQR 0.66–0.95] mg/dl, serum 2 0.40 [IQR 0.33–0.46] mg/dl) than in the control group (serum 1 0.85 [IQR 0.69 − 0.99] mg/dl, serum 2 0.48 [IQR 0.38 − 0.59] mg/dl) (Fig. 3). The same finding was observed for BUN levels in the IUGR group (serum 1 30.6 [IQR 21.6 − 41.6] mg/dl, serum 2 11.9 [IQR 8.4–15.6] mg/dl) compared to the control group (serum 1 47.2 [IQR 33.9–64.4] mg/dl, serum 2 19.8 [IQR 12.6–26.6] mg/dl) (Fig. 3). After Bonferroni correction, the results remained significant (*p* < 0.0125) for SCr and BUN collected between the 30th and 120th hour of life as well as for SCr collected between the 5th and 53rd day of life. This was confirmed by an analysis of covariance controlling for the highly variable timing of the second blood sampling as a confounder. Compared by *χ*^2^ testing with Bonferroni correction, participants with IUGR did not exceed normal values for SCr and BUN more often than the control group (Online Resource 2). Urine collection was successful in 87/99 participants (34/39 IUGR, 53/60 control). In 32 of these 87 neonates (8 IUGR, 24 control), a urine sample could be obtained between the 36th and 72nd hour of life. There was no significant difference in protein/creatinine ratio between cases and controls for albumin (*p* = 1), total protein (*p* = 0.332), α-1-microglobulin (*p* = 1), immunoglobulin G (*p* = 1) and transferrin (*p* = 1). Except for transferrin, for which no neonatal reference values are available, the respective specific limits for urinary excretion of the above parameters were not exceeded significantly more often by the IUGR neonates compared with the control group (Online Resource 2).

### Protein intake

There was no significant difference (*p* = 0.504) in protein supply between the groups during the first 30 to 120 h of life (IUGR 1.9 [IQR 1.4–2.5] g/kg body weight; controls 1.8 [IQR 1.2–2.4] g/kg body weight). At the time of the second serum measurement (serum 2), the protein intake was significantly higher (*p* = 0.01) in the IUGR group (3.8 [IQR 2.8–4.7] g/kg body weight) compared to controls (3.3 [IQR 2.6–3.9] g/kg body weight).

## Discussion

In our study, we did not observe any significant differences in blood pressure values and urinary protein/creatinine ratios between both groups. Significant differences existed in SCr levels at both measurement times and BUN at the first measurement (between the 30th and 120th hour of life), with higher concentrations in the control group.

IUGR is under investigation as a possible risk factor for kidney damage and high blood pressure in later life. Our aim was to detect early deviations in standard clinical and laboratory parameters between IUGR and non-IUGR neonates and young infants, which are in line with the observations of associated changes in kidney anatomy, nephrogenesis and the vascular system in other studies [14–16]. To our knowledge, few studies exist that specifically analyse the influence of IUGR in the neonatal and early infant period based on clinical parameters. Other studies have observed increased protein excretion or microalbuminuria and increased systolic blood pressure in IUGR infants [30, 31]. We could not reproduce these observations in our study, in which we examined the parameters even earlier postnatally.

Our recorded lower levels of BUN might be explained by a lower liver mass in IUGR patients [32]. This might also apply for the differences in SCr, as not only muscle mass and kidney function determine SCr levels, but also it is dependent on liver mass and function, as creatine, which is metabolised to creatinine, is produced there. The more frequent use of vancomycin, catecholamines and glucocorticoids in the first days of life is likely to be attributable to the higher morbidity associated with IUGR [8 − 11]. It seems that blood pressure measurements and examination of proteinuria in the neonatal and early infant period are not suitable to detect possible changes in kidney function, nephrogenesis and the vascular system due to IUGR, or it could suggest the hypothesis that there are no changes due to IUGR.

For further investigation based on our study results, it would be useful to expand the examined parameters, e.g., by adding cystatin C, angiotensin II, endothelin-1, fibroblast growth factor 23 and cathepsin B or even investigating the total proteome. In addition, an increase in the number of study participants, as well as long-term follow-up, would also be reasonable pursuits.

There is still a lack of fundamental studies that look closely at the possible early postnatal effects of IUGR, as often only generally low birth weight (LBW), small for gestational age (SGA) or prematurity has been investigated. It is of great importance to identify a risk for kidney damage as early as possible to initiate preventive steps before kidney impairment occurs. The investigation of routine parameters as a first step seems especially essential to us, as these parameters are determined during follow-up visits of at-risk children, following current recommendations [33]. Our study demonstrating no difference between IUGR and control children regarding these routine parameters warrants further studies that might lead to a method of early stratification of at-risk infants.

Certainly, our study design has some limitations that cannot be ignored. The more frequent occurrence of morbidities associated with IUGR could lead to a bias in the comparability of our groups. Numerous drugs, which were used both in IUGR and control patients, are classified as nephrotoxic or potentially nephrotoxic, though the exact dose-toxicity relationship is not completely clear, especially regarding neonates and young infants [34, 35]. It should be noted that sampling was performed in a very wide time window and at different times of day. As proteinuria is subject not only to a circadian rhythm but also to hydration status, an invasive 24-h urine sample would have been the gold standard but is too invasive to routinely perform in otherwise healthy neonates. Nevertheless, we tried to control for hydration status by normalising urine metabolites by creatinine excretion. During the first 72 h of life, neonatal SCr concentration is mainly dependent on maternal creatinine clearance. This affects the comparability during our first measurement time point, as 81/99 samples (29/39 IUGR, 52/60 control) were collected within the first 72 h of life. Unfortunately, several data points from study participants are missing, especially for the second serum measurement and the blood pressure values. This reduces the already small cohort and increases the influence of outliers. For laboratory values below the respective level of detection, we have assumed a value just below that limit for further calculations. Except for transferrin, only one value was below or above the LOD, so the bias caused by this procedure is almost negligible [36]. In the special case of transferrin, more than half of all values were below the LOD (42/73), as this corresponds to the physiological concentration of transferrin in urine [37]. Therefore, an interference of the result by the substitution of a constant value cannot be ruled out.

In summary, our study did not identify any differences in the clinical routine parameters investigated that indicate higher blood pressure or worse kidney function due to IUGR. Further studies including more specific parameters are needed to hopefully identify newborns and infants at-risk for future changes in kidney function.

## Supplementary Information

Below is the link to the electronic supplementary material.

Graphical Abstract (PPTX 68 KB)

Supplementary file2 (DOCX 20 KB)

Supplementary file3 (DOCX 18 KB)

## Declarations

This study was performed in accordance with the principles of the Declaration of Helsinki. Approval was granted by the local Ethics Committee of the University Bonn Medical School (22.05.2020/No. 079/19). Written informed consent was obtained from the parents or guardians. Parents or guardians signed informed consent regarding publishing their children’s data.

## References

[1] Albu A, Anca A, Horhoianu V, Horhoianu I (2014). Predictive factors for intrauterine growth restriction. J Med Life.

[2] Parker SE, Werler MM (2014). Epidemiology of ischemic placental disease: a focus on preterm gestations. Semin Perinatol.

[3] Mayer C, Joseph KS (2013). Fetal growth: a review of terms, concepts and issues relevant to obstetrics. Ultrasound Obstet Gynecol.

[4] Intrauterine growth restriction. Guideline of the German Society of Gynecology and Obstetrics (S2k, AWMF-Registry-No.: 015/080, 2016). http://www.awmf.org/leitlinien/detail/II/015-080.html. Accessed 6 Nov 2018 10.1055/s-0043-118908 PMC5784232 29375144

[5] Lees CC, Stampalija T, Baschat AA, Silva Costa F, Ferrazzi E, Figueras F, Hecher K, Kingdom J, Poon LC, Salomon LJ, Unterscheider J (2020). ISUOG Practice Guidelines: diagnosis and management of small-for-gestational-age fetus and fetal growth restriction. Ultrasound Obstet Gynecol.

[6] Melamed N, Baschat A, Yinon Y, Athanasiadis A, Mecacci F, Figueras F, Berghella V, Nazareth A, Tahlak M, McIntyre HD, Da Silva CF, Kihara AB, Hadar E, McAuliffe F, Hanson M, Ma RC, Gooden R, Sheiner E, Kapur A, Divakar H, Ayres-de-Campos D, Hiersch L, Poon LC, Kingdom J, Romero R, Hod M (2021). FIGO (international Federation of Gynecology and obstetrics) initiative on fetal growth: best practice advice for screening, diagnosis, and management of fetal growth restriction. Int J Gynecol Obstet.

[7] McCowan LM, Figueras F, Anderson NH (2018). Evidence-based national guidelines for the management of suspected fetal growth restriction: comparison, consensus, and controversy. Am J Obstet Gynecol.

[8] Frøen JF, Gardosi JO, Thurmann A, Francis A, Stray-Pedersen B (2004). Restricted fetal growth in sudden intrauterine unexplained death. Acta Obstet Gynecol Scand.

[9] Richardus JH, Graafmans WC, Verloove-Vanhorick SP, Mackenbach JP, EuroNatal International Audit Panel; EuroNatal Working Group (2003). Differences in perinatal mortality and suboptimal care between 10 European regions: results of an international audit. BJOG.

[10] McIntire DD, Bloom SL, Casey BM, Leveno KJ (1999). Birth weight in relation to morbidity and mortality among newborn infants. N Engl J Med.

[11] Alkalay AL, Graham JM, Pomerance JJ (1998). Evaluation of neonates born with intrauterine growth retardation: review and practice guidelines. J Perinatol.

[12] Pallotto EK, Kilbride HW (2006). Perinatal outcome and later implications of intrauterine growth restriction. Clin Obstet Gynecol.

[13] Barker DJP (2006). Adult consequences of fetal growth restriction. Clin Obstet Gynecol.

[14] Woods LL, Ingelfinger JR, Nyengaard JR, Rasch R (2001). Maternal protein restriction suppresses the newborn renin-angiotensin system and programs adult hypertension in rats. Pediatr Res.

[15] Skilton MR, Evans N, Griffiths KA, Harmer JA, Celermajer DS (2005). Aortic wall thickness in newborns with intrauterine growth restriction. Lancet.

[16] Hinchliffe SA, Lynch MR, Sargent PH, Howard CV, Van Velzen D (1992). The effect of intrauterine growth retardation on the development of renal nephrons. Br J Obstet Gynaecol.

[17] Brenner BM, Chertow GM (1994). Congenital oligonephropathy and the etiology of adult hypertension and progressive renal injury. Am J Kidney Dis.

[18] Wlodek ME, Westcott K, Siebel AL, Owens JA, Moritz KM (2008). Growth restriction before or after birth reduces nephron number and increases blood pressure in male rats. Kidney Int.

[19] Vehaskari VM, Aviles DH, Manning J (2001). Prenatal programming of adult hypertension in the rat. Kidney Int.

[20] Moritz KM, Mazzuca MQ, Siebel AL, Mibus A, Arena D, Tare M, Owens JA, Wlodek ME (2009). Uteroplacental insufficiency causes a nephron deficit, modest renal insufficiency but no hypertension with ageing in female rats. J Physiol.

[21] Pejovic B, Peco-Antic A, Marinkovic-Eric J (2007). Blood pressure in non-critically ill preterm and full-term neonates. Pediatr Nephrol.

[22] Rudd PT, Hughes EA, Placzek MM, Hodes DT (1983). Reference ranges for plasma creatinine during the first month of life. Arch Dis Child.

[23] Safer Care Victoria. Normal laboratory values for neonates. https://www.bettersafercare.vic.gov.au/clinical-guidance/neonatal/normal-laboratory-values-for-neonates. Accessed 23 Dec 2021

[24] Ponthier L, Trigolet M, Chianea T, Mons F, Yardin C, Guigonis V, El Hamel C (2021). Distribution of proteinuria- and albuminuria-to-creatinine ratios in preterm newborns. Pediatr Nephrol.

[25] El Hamel C, Chianea T, Thon S, Lepichoux A, Yardin C, Guigonis V (2017). Normal values of urine total protein- and albumin-to-creatinine ratios in term newborns. Pediatr Nephrol.

[26] Lehrnbecher T, Greissinger S, Navid F, Pfüller H, Jeschke R (1998). Albumin, IgG, retinol-binding protein, and alpha1-microglobulin excretion in childhood. Pediatr Nephrol.

[27] The R Project for Statistical Computing. https://www.R-project.org/. Accessed 28 Dec 2021

[28] Voigt M, Rochow N, Schneider KT, Hagenah HP, Scholz R, Hesse V, Wittwer-Backofen U, Straube S, Olbertz D (2014). Neue Perzentilwerte für die Körpermaße neugeborener Einlinge: Ergebnisse der deutschen Perinatalerhebung der Jahre 2007–2011 unter Beteiligung aller 16 Bundesländer [New percentile values for the anthropometric dimensions of singleton neonates: analysis of perinatal survey data of 2007–2011 from all 16 states of Germany]. Z Geburtshilfe Neonatol.

[29] Nada A, Bonachea EM, Askenazi DJ (2017). Acute kidney injury in the fetus and neonate. Semin Fetal Neonatal Med.

[30] Aisa MC, Cappuccini B, Barbati A, Orlacchio A, Baglioni M, Di Renzo GC (2016). Biochemical parameters of renal impairment/injury and surrogate markers of nephron number in intrauterine growth-restricted and preterm neonates at 30–40 days of postnatal corrected age. Pediatr Nephrol.

[31] Zanardo V, Fanelli T, Weiner G, Fanos V, Zaninotto M, Visentin S, Cavallin F, Trevisanuto D, Cosmi E (2011). Intrauterine growth restriction is associated with persistent aortic wall thickening and glomerular proteinuria during infancy. Kidney Int.

[32] Pendleton AL, Wesolowski SR, Regnault TRH, Lynch RM, Limesand SW (2021). Dimming the powerhouse: mitochondrial dysfunction in the liver and skeletal muscle of intrauterine growth restricted fetuses. Front Endocrinol.

[33] Iacobelli S, Guignard JP (2022) When the progresses in neonatology lead to severe congenital nephron deficit: is there a pilot in the NICU? Pediatr Nephrol 37:1277–1284. 10.1007/s00467-021-05338-8 10.1007/s00467-021-05338-8 34761299

[34] Stoops C, Stone S, Evans E, Dill L, Henderson T, Griffin R, Goldstein SL, Coghill C, Askenazi DJ (2019). Baby NINJA (Nephrotoxic Injury Negated by Just-in-Time Action): reduction of nephrotoxic medication-associated acute kidney injury in the neonatal intensive care unit. J Pediatr.

[35] Lefebvre CE, Filion KB, Reynier P, Platt RW, Zappitelli M (2020). Primary care prescriptions of potentially nephrotoxic medications in children with CKD. Clin J Am Soc Nephrol.

[36] Croghan C, Egeghy PP (2003) Methods of dealing with values below the limit of detection using SAS. Presented at Southeastern SAS User Group, St. Petersburg, FL, September 22–24, 2003. https://cfpub.epa.gov/si/si_public_record_report.cfm?Lab=NERL&dirEntryId=64046

[37] Rifai N, Gubar K, Silverman LM (1987). Immunoturbidimetry: an attractive technique for the determination of urinary albumin and transferrin. Clin Biochem.

