# Supplementary material for: Blood pressure and kidney function in neonates and young infants with intrauterine growth restriction
Source: Pediatr Nephrol. 2022 Sep 2;38(4):1223–32. doi: 10.1007/s00467-022-05713-z (PMC9925571; doi:10.1007/s00467-022-05713-z)
Supplement: Supplementary file 1 — Graphical Abstract (PPTX 68 KB) [file 467_2022_5713_MOESM1_ESM.pptx]

## Slide 1
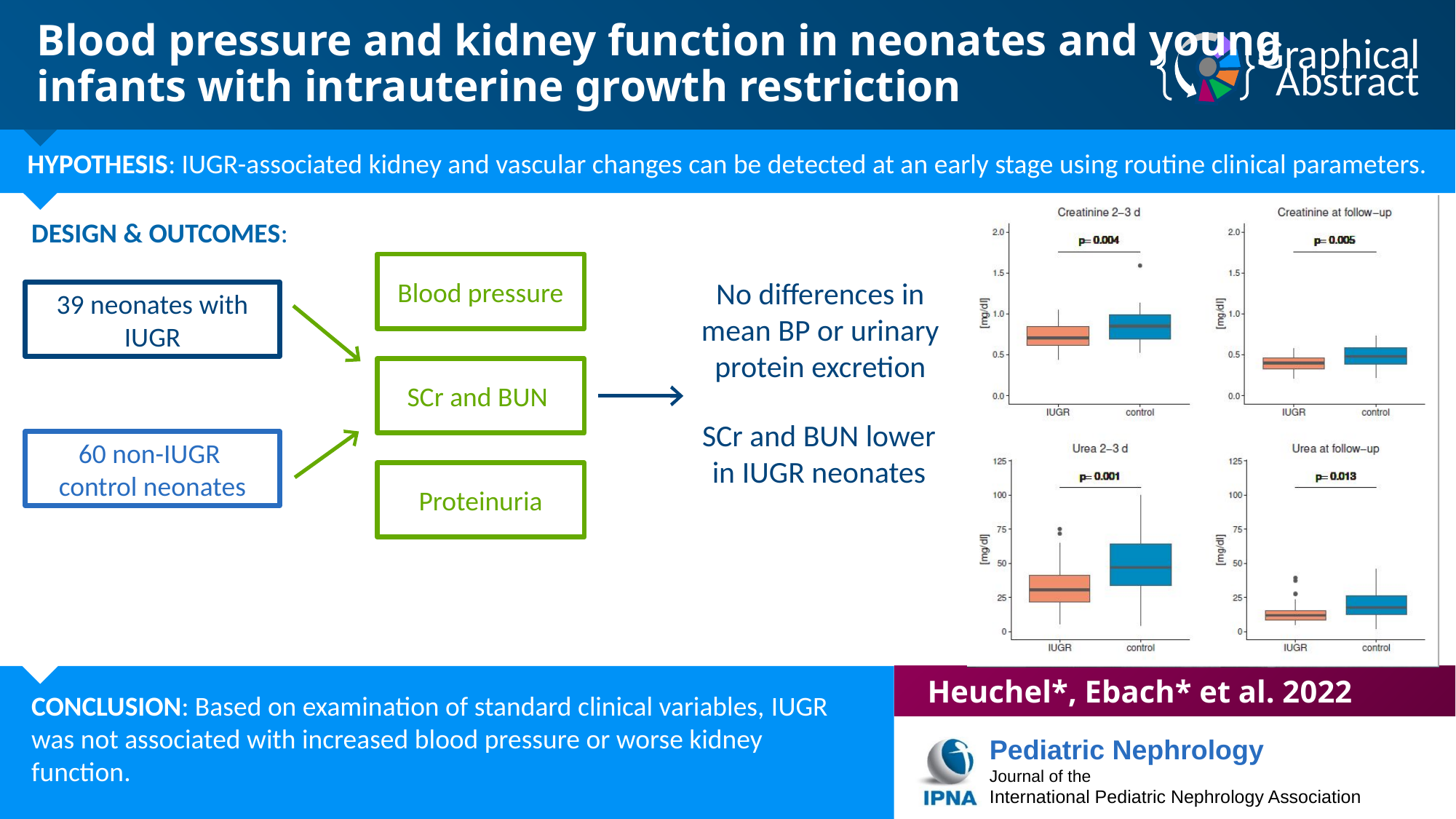

Blood pressure and kidney function in neonates and young
infants with intrauterine growth restriction
HYPOTHESIS: IUGR-associated kidney and vascular changes can be detected at an early stage using routine clinical parameters.
DESIGN & OUTCOMES:
Blood pressure
No differences in mean BP or urinary protein excretion
39 neonates with IUGR
SCr and BUN
SCr and BUN lower in IUGR neonates
60 non-IUGR
control neonates
Proteinuria
Heuchel*, Ebach* et al. 2022
CONCLUSION: Based on examination of standard clinical variables, IUGR was not associated with increased blood pressure or worse kidney function.
