# Supplementary material for: Blood pressure and kidney function in neonates and young infants with intrauterine growth restriction
Source: Pediatr Nephrol. 2022 Sep 2;38(4):1223–32. doi: 10.1007/s00467-022-05713-z (PMC9925571; doi:10.1007/s00467-022-05713-z)
Supplement: Supplementary file 2 — Supplementary file2 (DOCX 20 KB) [file 467_2022_5713_MOESM2_ESM.docx]

|  | IUGR | control | P overall |
| --- | --- | --- | --- |
| BP 1 |  |  |  |
| systolic [mmHg] | 57.5 ± 10.9 (n = 36) | 60.9 ± 8.4 (n = 59) | 0.109 |
| mean z-score | 0.8 ± 1.6 | 1.1 ± 1.6 |  |
| MAP [mmHg] | 44.1 ± 8.3 (n = 36) | 46.2 ± 6.6 (n = 59) | 0.202 |
| mean z-score | 0.6 ± 1.5 | 0.7 ± 1.5 |  |
| diastolic [mmHg] | 35.4 ± 7.8 (n = 36) | 36.5 ± 6.5 (n = 59) | 0.479 |
| mean z-score | 0.1 ± 1.4 | 0.1 ± 1.4 |  |
| BP 2 |  |  |  |
| systolic [mmHg] | 63.0 ± 8.5 (n = 35) | 64.1 ± 7.9 (n = 49) | 0.547 |
| mean z-score | 1.0 ± 3.0 | 0.6 ± 1.4 |  |
| MAP [mmHg] | 47.6 ± 6.8 (n = 35) | 47.3 ± 7.4 (n = 49) | 0.851 |
| mean z-score | 0.3 ± 1.9 | 0.1 ± 1.7 |  |
| diastolic [mmHg] | 38.6 ± 7.1 (n = 35) | 38.4 ± 7.1 (n = 49) | 0.938 |
| mean z-score | −0.2 ± 1.9 | −0.3 ± 1.6 |  |
| BP 3 |  |  |  |
| systolic [mmHg] | 71.5 ± 12.8 (n = 36) | 69.6 ± 8.2 (n = 58) | 0.420 |
| mean z-score | 1.2 ± 4.0 | 0.3 ± 2.3 |  |
| MAP [mmHg] | 50.3 ± 8.9 (n = 36) | 50.7 ± 7.0 (n = 58) | 0.823 |
| mean z-score | −1.0 ± 2.1 | −0.8 ± 1.7 |  |
| diastolic [mmHg] | 37.6 ± 7.2 (n = 36) | 39.7 ± 7.1 (n = 58) | 0.160 |
| mean z-score | −2.1 ± 1.5 | −1.4 ± 1.7 |  |

**Online Resource 1** Arterial blood pressure at different time points presented as means with standard deviation. The p-values refer to a two-tailed T-Test comparing IUGR and control patients. Z-scores were adapted from Pejovic B, Peco-Antic A, Marinkovic-Eric J (2007) Blood pressure in non-critically ill preterm and full-term neonates. Pediatr Nephrol 22:249–257. https://doi.org/10.1007/s00467-006-0311-3

IUGR, intrauterine growth restriction; BP, blood pressure; MAP, mean arterial pressure
