# Supplementary material for: Blood pressure and kidney function in neonates and young infants with intrauterine growth restriction
Source: Pediatr Nephrol. 2022 Sep 2;38(4):1223–32. doi: 10.1007/s00467-022-05713-z (PMC9925571; doi:10.1007/s00467-022-05713-z)
Supplement: Supplementary file 3 — Supplementary file3 (DOCX 18 KB) [file 467_2022_5713_MOESM3_ESM.docx]

|  | IUGR | control | p. overall |
| --- | --- | --- | --- |
| first SCr^1^ |  |  |  |
| above upper normal value | 0 | 1 (2%) | 1* |
| below lower normal value | 13 (34%) | 14 (24%) | 0.372 |
| second SCr^2^ below lower normal value | 5 (18%) | 4 (10%) | 0.468* |
| first BUN^1^ |  |  |  |
| above upper normal value | 2 (5%) | 11 (19%) | 0.107 |
| below lower normal value | 5 (13%) | 6 (10%) | 0.748* |
| second BUN^2^ below lower normal value | 21 (75%) | 19 (45%) | 0.027 |
| urine protein excretion^3^ above upper normal value |  |  |  |
| total protein | 9 (26%) | 8 (15%) | 0.304 |
| albumin | 5 (15%) | 4 (8%) | 0.304* |
| α-1-microglobulin | 8 (24%) | 8 (15%) | 0.479 |
| immunoglobulin G | 9 (26%) | 13 (25%) | 1 |

**Online Resource 2** Exceeding specific limits for SCr, BUN and urine parameters. Using Pearson’s Chi-squared test with Yates’ continuity correction**.** IUGR, intrauterine growth restriction; SCr, serum creatinine; BUN, blood urea nitrogen.

**^1^** 30^th^−120^th^ hour of life; ^2^ 5^th^−53^rd^ day of life; ^3^ 30^th^−240^th^ hour of life

***** In case of expected frequencies < 5 Fisher’s exact test was applied

Reference values by Ponthier L, Trigolet M, Chianea T et al (2021) Distribution of proteinuria- and albuminuria-to-creatinine ratios in preterm newborns. Pediatr Nephrol 36**:**1515–1524. https://doi.org/10.1007/s00467-020-04838-3; El Hamel C, Chianea T, Thon S et al (2017) Normal values of urine total protein- and albumin-to-creatinine ratios in term newborns. Pediatr Nephrol 32:113–118. https://doi.org/10.1007/s00467-016-3427-0; Lehrnbecher T, Greissinger S, Navid F et al (1998) Albumin, IgG, retinol-binding protein, and α_1_-microglobulin excretion in childhood. Pediatr Nephrol 12**:**290–292. https://doi.org/10.1007/s004670050455; Rudd PT, Hughes EA, Placzek MM, Hodes DT (1983) Reference ranges for plasma creatinine during the first month of life. Archives of Disease in Childhood 58:212-215. https://doi.org/10.1136/adc.58.3.212; Victorian Agency for Health Information (2013) Normal laboratory values for neonates. Safer Care Victoria. https://www.bettersafercare.vic.gov.au/clinical-guidance/neonatal/normal-laboratory-values-for-neonates. Accessed 13 July 2021
